# Flipons enable genomes to learn by intermediating the exchange of energy for information

**DOI:** 10.1098/rsif.2025.0049

**Published:** 2025-03-26

**Authors:** Alan Herbert

**Affiliations:** ^1^ Discovery, InsideOutBio Inc, Charlestown, MA, USA

**Keywords:** DNA topology, flipons, entropy, learning, genome, evolution

## Abstract

Recent findings have confirmed the long-held belief that alternative DNA conformations encoded by genetic elements called flipons have important biological roles. Many of these alternative structures are formed by sequences originally spread throughout the human genome by endogenous retroelements (ERE) that captured 50% of the territory before being disarmed. Only 2.6% of the remaining DNA codes for proteins. Other organisms have instead streamlined their genomes by eliminating invasive retroelements and other repeat elements. The question arises, why retain any ERE at all? A new synthesis suggests that flipons enable genomes to learn and programme the context-specific readout of information by altering the transcripts produced. The exchange of energy for information is mediated through changes in DNA topology. Here I provide a formulation for how genomes learn and describe the underlying p-bit algorithm through which flipons are tuned. The framework suggests new strategies for the therapeutic reprogramming of cells.

## 1. Introduction

The role in biology of alternative DNA conformation like Z-DNA, G-quadruplexes (GQs), triple helices, i-motifs and others less well defined has until recently been controversial. As recently as 2007, searching for a biological Z-DNA was dismissed as ‘a dead end’ pursuit [1]. A host of recent findings have confirmed essential roles for Z-DNA and Z-RNA (collectively called ZNAs) in regulating innate immunity against viruses and cancers, and autoimmune responses [2–6]. The supporting evidence derives from many disciplines, including the detailed studies by structural, physical and biological chemists, the functional mapping of pathways by cellular biologists, and their genetic validation in both human and rodent studies [2,7–14]. Other roles for Z-DNA in transcription are also supported by computational and biological studies [15–20]. It is now possible to trace the Zα domain that binds Z-DNA [21], Z-RNA and GQ [22] back to the earliest eukaryotic unicellular precursor *Capsaspora owczarzaki* [15].

This work has led to the concept of a flipon, a genetically encoded element that regulates outcomes by its shape rather than by sequence [23,24]. The switch to an alternative fold requires energy that can arise from changes in local supercoiling due to the action of processive enzymes such as helicases and polymerases or the release of proteins such as histones that wrap DNA around themselves [25]. By tuning flipon conformation, the phase transitions associated with the readout of genetic information can operate close to criticality to vary the transcripts produced. In this review, I provide a formulation of how genomes learn to optimize flipon states. The loss function is based on well-established statistical principles that minimize the free energy costs incurred. The framework suggests new strategies for therapeutically reprogramming cells.

## 2. Flipons come in different shapes

There are many different types of flipon, each with a characteristic repeat sequence. For example, runs of guanosine can hydrogen-bond with each other to form tetrads that stack into GQs [26]. Alternating purine, pyrimidine repeats can invert the base pairs to form left-handed Z-DNA [27], while direct purine and pyrimidine repeats can form triplexes [28–30]. Cytosine base pairs can also intercalate to form i-motifs [31].The energy required to initiate the transition can be generated within the cell by polymerases and helicases, or by other sources of helical stress, such as stretching and twisting [32,33].

The spread of flipons throughout genomes has largely been driven by transposons, with retrotransposition playing an important role in vertebrates. In humans, the ALU elements are usually dimeric and ~280 bases long These repeats were named for the *Arthrobacter luteus* restriction endonuclease that cuts ALUs in genomic DNA at positions 168 and 228 to produce 49 base pair fragments. ALUs comprise about 11% of the total genomic sequence. They activate innate immunity by forming Z-RNA s. ALUs also contain GQ and triplex sequence motifs with biological activity [34], as do the long interspersed nuclear elements and long terminal repeat (LTRs) comprising another 20% of the human genome [35]. The high frequency of flipons in the genome renders them uninformative as B-DNA. However, in their alternative conformation, flipons flag active parts of the genome for the cellular machinery to engage. As flipon sequences are embedded in DNA and therefore heritable, they are subject to natural selection. In the simplest form, the encoding is binary, with flipons existing in either one shape or another. The flip occurs without any alteration to the underlying DNA sequence and without requiring strand breakage. For many situations, this outcome works well, with the switching on or off of innate immune responses by Z-DNA or Z-RNA providing an example [3–6]. However, many repeat sequences can adopt more than one alternative conformation. For example, the conformation adopted by a d(G:C)_n_ repeat B-DNA varies with its length: short duplex repeats are capable of forming Z-DNA [36], while longer stretches not only fold into GQs in single-stranded regions of DNA [37], but can accommodate a third strand to become triplex [38]. Further, the complementary cytosine strand can assemble into an i-motif [31]. The conformation adopted by d(G:C)_n_ repeat B DNA can vary with the location: triplex formation is highest in ribosomal arrays at sites where the meiotic recombination protein PRDM9 binds [39], with Z-DNA favoured downstream of a promoter [40,41] and GQ formation by oxidative damage to the guanine base [42]. Base modifications also alter the energetic cost of flipping other flipon types to an alternative conformation, with the d(^5^meC-G)_n_ forming Z-DNA under physiological salt conditions [43].

The rather dynamic formation of ZNA compared with the more stable nature of GQ and other flipons accounts for differences in the biology of these different flipon types. For example, during transcription, Z-flipons allow for the rapid reset of promoters. By contrast, G-flipons depend more on a lock and load mechanism, where GQ resolution is triggered by specific events. This mechanism times the onset of transcription, ensuring that promoters only fire when tripped [44–50]. The various roles for Z- and G-flipons in modulating transcription are well known and have been recently reviewed [50–53].

## 3. The evolutionary framework

**Figure 1.**
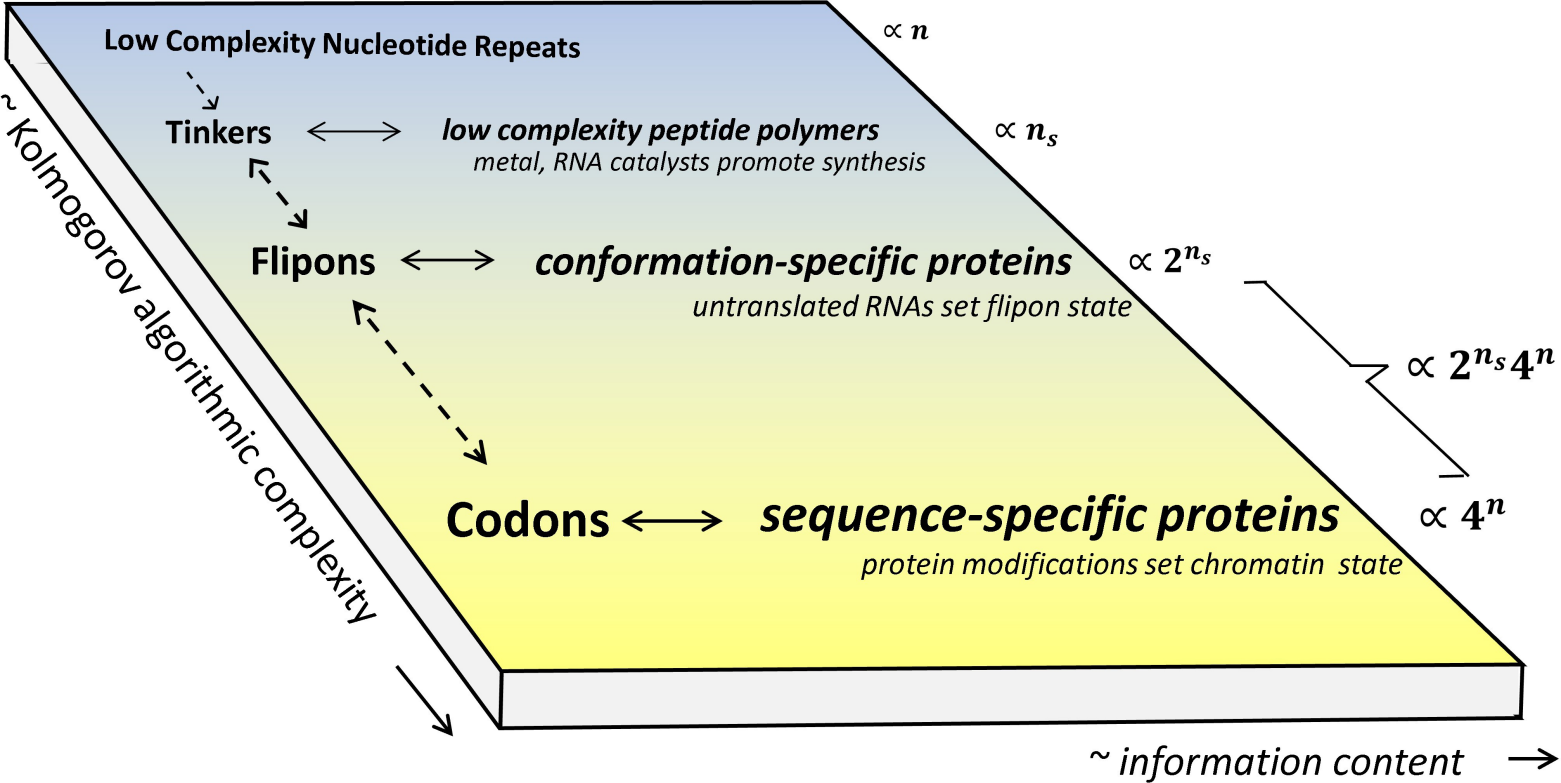
The evolutionary landscape of genetic regulation. A two-dimensional view of the increase in informational content and computational complexity over time, with tinkers as the first self-replicating agents that probably contributed to the development of the genetic code. The different flipons denoted by *n*_s_ switch between two or more states with probability *p_s_*, with the conformation adopted depending on the length of the flipon, as discussed in the text. Flipons code by their shape, which, at an early stage, was probably regulated by sequence-specific interactions with other nucleic acids, rather than by the sequence-specific binding of proteins, which were a later innovation. The evolution of sequence-specific proteins provided another layer of regulation. Their synthesis was enabled once tinkers elaborated the genetic encoding of amino acids. The combination of codons and flipons greatly increased the coding potential of genomes as indicated by the scale on the right.

Questions arise about the origins of flipons and their fit with the current concepts of gene regulation. It is likely that flipon-based mechanisms arose before codons evolved (figure 1). At an early stage, simple nucleic repeats capable of self-replication emerged from the primordial soup. Given the harsh conditions, these polymers probably adopted many alternative conformations. Informational roles arose from their interactions with peptide polymers and metals, a subset of which were catalytic [54]. I have called these entities ‘tinkers’ in reference to François Jacob’s description of Nature as a tinkerer [55]. The shape and sequence-specific interactions essential for the self-replication of tinkers probably contributed to the modern genetic code. The base-specific interactions with other nucleic acids embedded catalytic tinkers and flipons within modern genomes [53,56].

The information content of the first tinkers was low due to their repetitive nature. Those sequences able to form non-B-DNA conformations were able to nucleate different complexes in response to environmental change. The structure-specific outcomes increased their adaptability. The subsequent evolution by tinkers of sequence-specific proteins exploited the much greater dimensionality of a four-base code compared with one based on the different flipon structures. Coding by base rather than by backbone yielded a greater diversity of outcomes. As genomes developed, duplication of genes exploited adaptations that were successful in the past, with mutations giving rise to new functionalities [57]. Nevertheless, the new system was built on the flipons that already existed. Clearly, adaptations that relied on flipons to protect against pathogens still persist until this day, although in some organisms they have been supplanted by small interfering RNAs that bind to pathogen nucleic acids in a sequence-dependent manner to disrupt their function [15]. The retention of flipons also proved advantageous in other ways. By varying how coding transcripts are expressed and processed, flipons increased the algorithmic complexity of the RNAs produced [58]. The fliponware enabled the encoding of alternative gene products without a change in the codonware [59]. The transcripts generated varied dynamically, allowing rapid adaptation as selection pressures changed.

Both flipons and codons therefore play an important but unique role in the evolution of new phenotypes. The two forms of genetic encoding optimize outcomes in a different manner. With codons, the need to maintain function restricts the sequence space that can be explored. The limitations are most severe for proteins that perform multiple functions, where a mutation that optimizes a particular outcome could compromise its other roles. As Ohno noted, one way to escape such severe strictures is to duplicate protein coding genes and evolve each copy separately, ensuring that the different functionalities are conserved in the genome [57]. Flipons evolve in different ways. They can explore a larger sequence space than codons and vary the protein products made by altering gene expression and RNA processing. By flipping to an alternative conformation, flipons can just simply capture the energy needed to maintain an open chromatin region, enabling a sequence-specific transcription factor (TF) to dock to a promoter or an enhancer element. Alternatively, any effect that prevents the flip can simply prevents expression of that RNA by impeding the maintenance of an open chromatin region. The spread of flipons throughout the genome by transposable elements also generates phenotypic variation by changing the readout of genetic information in the neighbourhood where the insertion occurs. When integrated into pathways that perform core cellular functions, flipons will exhibit phylogenetic sequence conservation, as recently shown [16]. Interestingly, Z-DNA is not, as once thought, mutagenic, and GQs are only mildly so, suggesting that mutation is not the primary way that flipons evolve [60].

**Figure 2.**
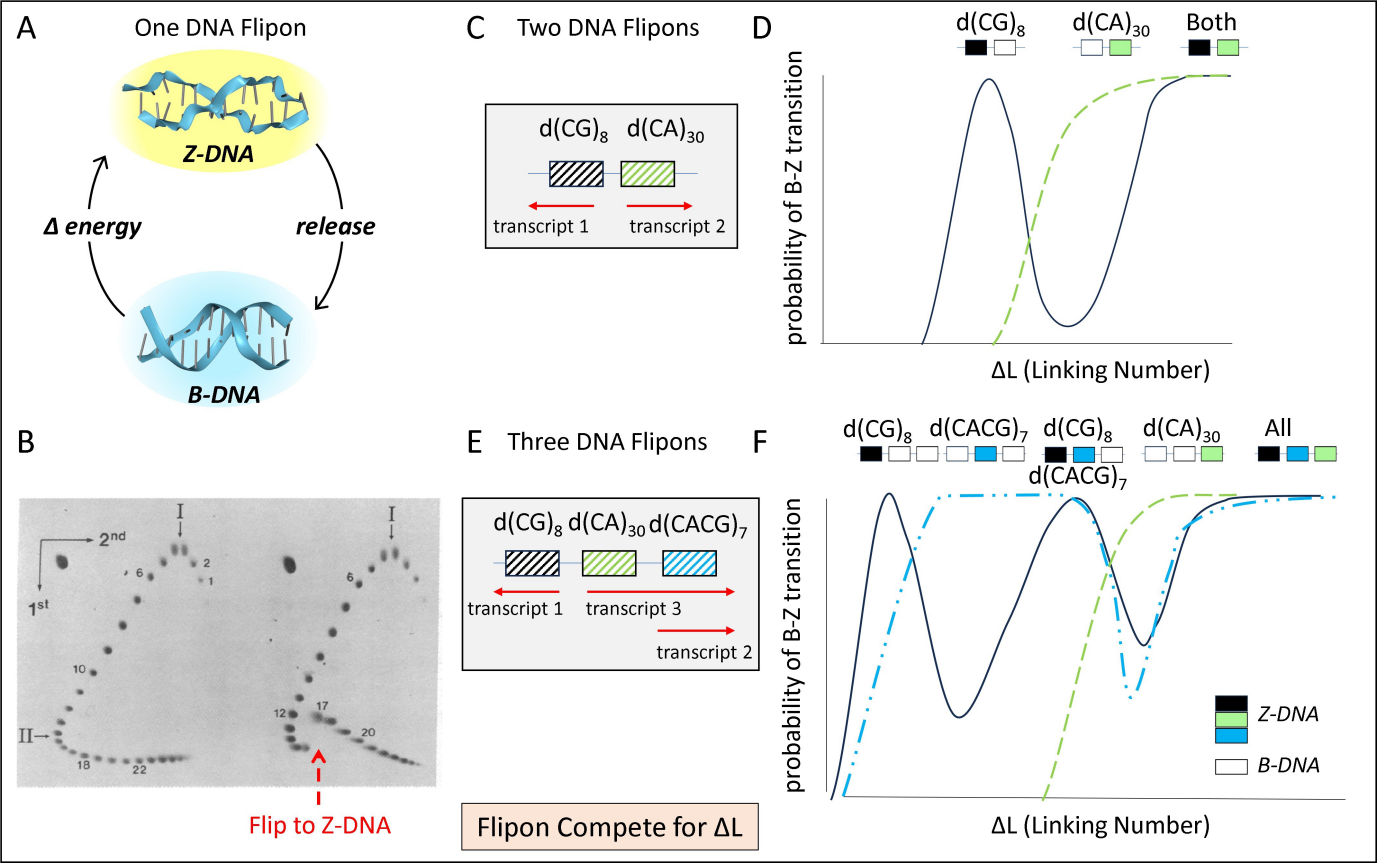
Flipons compete for energy. (A) The cycling of a Z-flipon between B-DNA and Z-DNA requires an input of energy that can be used to power different biological outcomes. (B) The change in linking number can be determined experimentally by separating topoisomers on two-dimensional agarose gels. A Z-DNA forming plasmid is shown on the right with the parental plasmid on the left. The change in twist associated with Z-DNA formation by the d(CG)_16_ insert can be determined by comparing topoisomers 12 and 17 (as labelled in panel (B)), which have equivalent writhe, i.e. Δ*L*= Δ*T* (from [64] with permission of James Wang). (C) Two adjacent flipons (indicated by the striped boxes) that are transcribed in opposite directions as indicated by the red arrows (adapted from [65]). (D) The conformation of each flipon depends on the level of negative supercoiling and the conformation of the other. The black shading indicates Z-DNA formation while the white and striped boxes indicates B-DNA, showing how d(CG)_8_ flips to Z-DNA, back to B-DNA and then again to Z-DNA, then back to B-DNA and finally to Z-DNA again. (E,F) The three adjacent flipons that are transcribed in opposite directions. The set of active flipons changes as the negative supercoiling increases, with five different combinations shown (adapted from [66]).

Flipon conformation is programmable with RNAs, both long and short. For example, the formation of triplexes induced by long non-coding RNAs (lncRNAs) has been experimentally demonstrated in a number of systems. Evidence also exists for the programmability of G- and Z-flipon conformation by RNAs ([30,61–63] and [56] plus reference within) that bind flipons in a sequence-specific manner (figure 2). Such interactions allow the use of miRNAs and other small RNAs such as tRNA fragments to regulate tissue development and to target flipons throughout the genome selectively. The interactions constrain the sequence variation of both miRNAs and flipons.

Selection pressure on flipons can also arise from transgenerational effects. Notably, suppression of flipon containing retroelements in the embryo by small sequence-specific piRNAs and t-RNA fragments is well known, as well as proteins such as ZBTB43 that target specifically a subset of Z-flipons composed of d(A–C) repeats [67–69]. However, small RNAs transmitted from the parents also potentially play a role in bootstrapping embryonic development [70–72]. By programming flipon conformation, the small RNAs help define the regions of open chromatin that are formed later in development, They probably act by placing epigenetic tags throughout the genome at the bivalent promoters later involved with tissue specification [56].

Exactly how flipon conformations were optimized during evolution is an interesting question, as it also relates to how genomes learn.

## 4. Flipons and codons: topology versus typography

The flipon-based systems work differently from the classical models of genetic programming, in which input sets a cell state that drives the response. This scheme is exemplified by the bacterial studies of Jacob and Monod, where an operon clusters the genes needed for the response to a particular environmental change [73]. By contrast, flipons settings are tasked with producing a particular state, either maintaining the current circuitry or switching to another programme. The system is extremely plastic, generating a variety of solutions, with some more robust than others. Interestingly, triplexes, Z- and G-flipons are clustered throughout the genome, particularly in promoter regions of genes where there are chromatin-free regions detectable by DNase footprinting [20,40,74–79]. This arrangement allows the conformation of flipons to vary, with many different combinations of flipon states possible. The exact promoter configuration depends on the local level of supercoiling (figure 2) and its partitioning between the different flipon types in the neighbourhood. These outcomes have been studied *in vitro* and *in vivo* [16,40,41,53,80,81].

*In vitro*, flipons are easily studied in isolation, allowing the determination of various parameters that determine their conformation (figure 2A). The literature for Z-DNA, G-quadruplex es, triplexes, and more recently, for i-motifs is quite extensive with the cellular biology of Z-DNA and G-quadruplexes best understood [45,82–84]. In the case of a single Z-flipon, the transition is quite straightforward as shown in figure 2B. Here, supercoiling is defined by the linking number *L*, which is the number of times the two DNA strands of the double helix twist around each other (*T* is positive for right-handed B-DNA and negative for left-handed Z-DNA), plus the writhe, which is the number of times a DNA crosses itself when imaged on a flat surface (*W* is positive for a right-handed cross). Defined this way, *L = T + W*. Normally, it is sufficient to measure the difference in *L (*Δ*L =* Δ*T +* Δ*W*) relative to a reference DNA segment. The values are ascertained using agarose gels to separate the different plasmid topoisomers at sufficient resolution to detect each unit change in *L*. Topoisomers that have a difference in *L* but the same *W*, are used to determine the change in *T* due to the formation of an alternative flipon conformation: since Δ*W =* 0 in this case, then Δ*L =* Δ*T* [64–66,85]. This is shown in figure 2B, where the topoisomers labelled 12 and 17 have the same writhe after the flip to Z-DNA as indicated by the red arrow, giving the Δ*T* for the d(CG)_16_ insert as equal to – 5 (corresponding to a change in helix winding of – (1/12+1/10)/base with 0.4 turns per B–Z junction). By comparing Z-DNA forming inserts of different sizes, it is possible to estimate the energetic cost of flipping (Δ*E*) and of forming B–Z junctions [86].

Techniques for estimating Δ*L* in cells after perturbation also exist [87–91]. The superhelical density is given by *σ* = Δ*L*/*N* (*n* = length of the closed circular DNA segment). In one system, the negative supercoiling upstream of a divergent, bidirectional, actively transcribing promoter can generate *σ* ≥ −0.07 [76,88]. This level is more than enough to flip a d(CG)_16_ insert to Z-DNA where a *σ* = −0.011 is sufficient. By comparison, a nucleosome reduces *σ* by approximately −0.0086 ((Δ*L* = −1.26)/147 bases) [90]. The critical *σ* value to flip a sequence can vary with modifications to the DNA bases. For example, methylation of cytosine lowers the energy needed to propagate the change in conformation to adjacent sequences [43], as do polyamines such as spermine [92]. Topoisomerases and polymerases can also alter the local superhelical density to regulate the energy available to power the flip [20,32].

The situation is more complex when neighbouring flipons compete for the available supercoiling. An example is proved by the *in vitro* analysis of a closed circular DNA segment that contains two Z-DNA forming segments that differ in length and in their propensity to flip to Z-DNA (figure 2C,D) [65]. The longer d(CA)_n_ flipon is less likely to form Z-DNA than the shorter d(CG)_n_ flipon, as more energy is required to initiate the initial transition. As negative supercoiling increases, the d(CG)_n_ sequence first forms Z-DNA. As the supercoiling increases, the d(CA)_n_ then flips. Due to the cooperativity of the flip, the d(CA)_n_ flipon absorbs all the available energy, eventually causing the d(CG)_n_ segment to flip back to B-DNA as there is insufficient -*σ* available to maintain the Z-DNA conformation. The situation is even more complicated when three flipons are connected, as in figure 2E,F. In the case of d(CG)_n_, the flip is reversed when d(CACG)_n_ forms Z-DNA and then again when d(CA)_n_ flips [66]. Each time d(CG)_n_ forms Z-DNA, a region of open chromatin forms (dark boxes in figure 2C–F indicate Z-DNA formation and active promoters, while striped boxes represent flipons in the B-DNA conformation), potentially facilitating transcription (as indicated in the figure by the direction of the red arrows). The promoter associated with d(CG)_n_ is then active either alone or in conjunction with one or both of the other two promoters, producing a different combination of transcripts.

The competition for energy from other flipons in the neighbourhood enables the reprogramming of cells, with multiple transitions between active and idle states as the level of free supercoiling increases. The resetting of flipons increases phenotypic diversity. The most extreme example of plasticity is the reversion of a mature cell to a stem cell state [93]. Interestingly, these processes appear to involve miRNAs and are inhibited in culture by knockout of either the *DICER1* or *DGCR8* genes [94]. Intriguingly, conserved miRNAs also have seed sequence binding sites in promoters that overlap flipons and the potential to regulate their conformation [56].

## 5. The statistical framework

**Figure 3.**
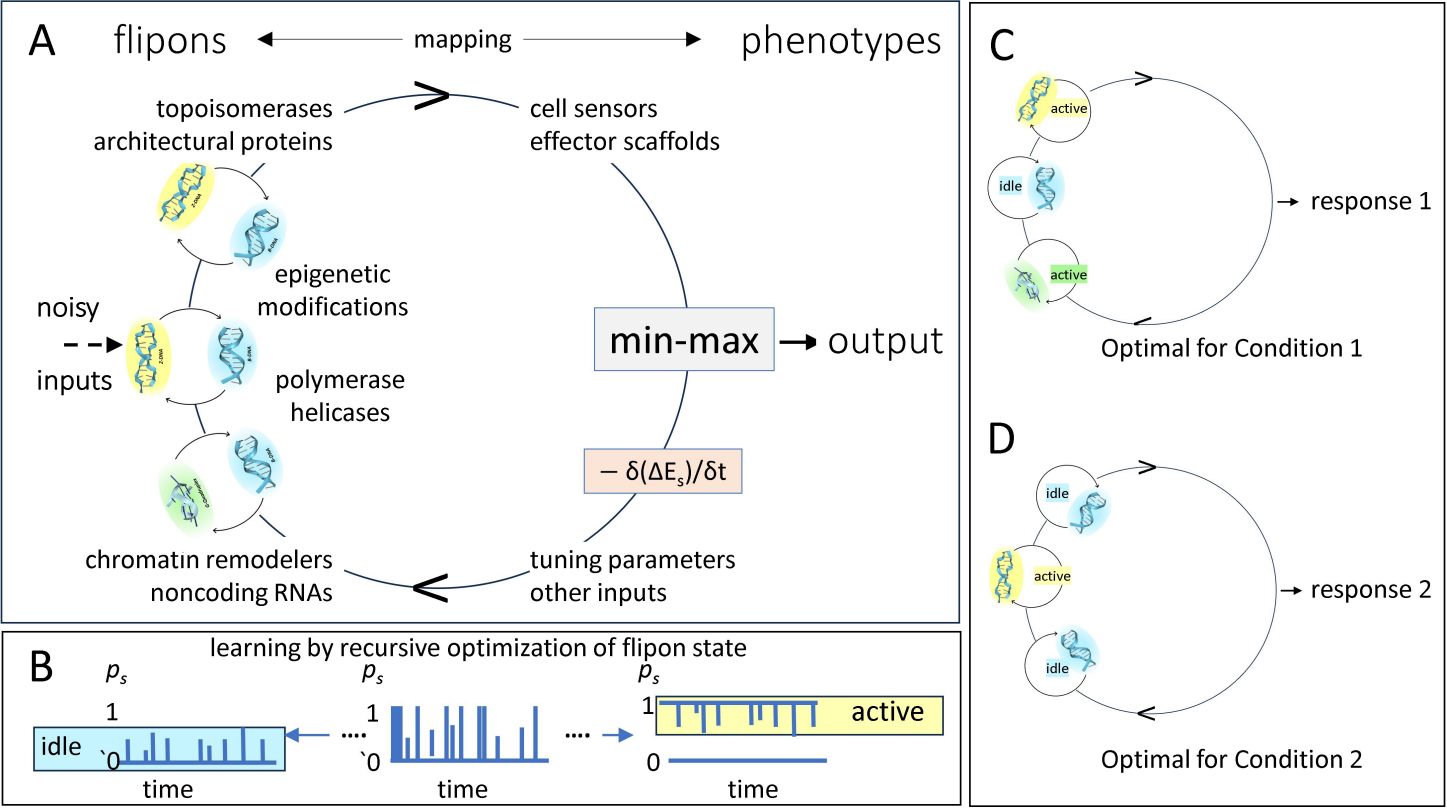
Flipon calibration. (A) Flipon settings are tuned to minimize or maximize a particular output in a variety of ways that impact the amount of energy necessary to induce the flip, and through pathways that control the local level of supercoiling. The process is reiterative (as indicated by the directed cycle) and optimizes the mapping of the flipon state to output. Initially, flipons cycle stochastically between B-DNA (coloured blue) and the non-B-DNA active conformation (coloured yellow for Z-DNA or green for GQ). (B) Learning alters the probability *p_s_* that a flipon will either be idle or active, as discussed in the text. (C,D) The phenotypes produced vary by condition with only some flipons cycling to the active state while the others remain idle.

Flipon-dependent outcomes are determined by how energy is partitioned between the different flipon states and each flipon type (figure 3). The transitions can be modelled using Jaynes’s principle of maximal entropy [95], which subsumes the Shannon generalization of the Gibbs formulation [96–98]. The approach selects the probability distribution with the greatest entropy by partitioning the available free energy of supercoiling between the alternative DNA conformations. The elaboration is information theoretic and assumes only the measurable features of a system.

The following description is a general formulation for the thermodynamic equilibria involved and is currently experimentally validated for simple systems based on covalently closed, circular plasmids, which have DNA ends ligated together to constrain supercoiling. The approach measures the effects of DNA sequence variants on the partitioning of superhelical stress within the plasmid and the energetic cost of flipping these DNA variants to an alternative conformation. In more complex systems, such as in genomes and cells, ends are usually fixed by anchoring to a scaffold, leading to the creation of topological domains [99].

The analysis starts with the familiar definition of the Helmholtz Free Energy F.


(5.1) F=⟨E⟩−TS,

where ⟨E⟩ is the estimate of the internal energy, *T* is the temperature in K. The aim is to find a partition function *Z* that gives the best fit to the data (figure 3B), assuming a Boltzmann distribution of flipon states where *n* = the number of states *S*, with *n*_0_ representing the count at the lowest energy state (defined as B-DNA for flipons), Δ*E*_s_ is the energy difference between the alternative flipon conformation and the ground state *E_0_* with S ≥ 1, s∈N, with


(5.2) $$N=\;n_{0}+n_{0}e^{-\Delta E_{1}/K_{B}T}+n_{0}e^{-\Delta E_{2}/K_{B}T}+\cdot \cdot \cdot n_{0}e^{-\Delta E_{s}/K_{B}T},$$

(5.3) $$Z=\frac{N}{n_{0}}=1+e^{-\Delta E_{1}/K_{B}T}+e^{-\Delta E_{2}/K_{B}T}+\cdot \cdot \cdot e^{-\Delta E_{s}/K_{B}T}$$

and the probability of each state after normalization by *Z* given by


(5.4) $$p_{s}=\frac{e^{-E_{s}/K_{B}T}}{Z},\;\;p_{s}\geq 0,$$

with the expected value of *E* and *S* after normalization given by


(5.5) $$\left\langle E\right\rangle =\;\sum_{s}E_{s},$$

(5.6) $$\left\langle S\right\rangle =\sum_{s}p_{s}ln\left(p_{s}\right),\;with\;\sum_{s}p_{s}=\sum_{s}\frac{e^{-\left(E_{s}\text{/}K_{B}T\right)}}{Z}=\;1.$$

Substitution of $p_{s}$ yields the relationship between entropy and the partition function


(5.7) $$\left\langle S\right\rangle =\frac{\left\langle E\right\rangle }{T}+K_{B}\ln\left(Z\right).$$

Given the change in linking number $\Delta L=\Sigma_{s}\left(\Delta L_{s}\right)$ is the upper bound of supercoiling, and with *T* the twist and *W* the writhe, then


(5.8) ΔL=ΔT+ΔW.

The measured ∆L is numerically related to the Δ*E* by


(5.9) $$\Delta E_{s}=w{\left(\Delta L_{s}\right)}^{2},$$

with w=1100RT/N [64,100], where R is universal gas constant, and N > 2000 bases [101]. The probability distribution of flipon states and their internal energy can then be estimated using the Lagrange multipliers *λ*, *μ* and *ν* using the specified constraints,


(5.10) $$L=\sum_{s}p_{s}\ln\left(p_{s}\right)-\lambda \sum_{s}p_{s}e^{-\left(E_{s}/K_{B}T\right)}+\mu \left(\sum_{s}p_{s}-1\right)+\nu \left(\Delta L-\sum_{s}\left(\Delta L_{s}\right)\right).$$

Solving L in the usual way yields a solution where all partial derivatives of L vanish. The first term for L equates to the Shannon entropy, the second term to the energy distribution across states, the third term to the probability of each structure and the last term provides the upper bound for supercoiling of the system. The optimal solution for L is usually found by enumeration. *In vitro* estimates of the energetic cost of nucleating the flip by creating a junction and for propagating a conformation to adjacent bases speed the calculation (figure 3B). Other costs vary by flipon type and arise from the concomitant compression, stretching and twisting of adjacent B-DNA regions that depend on the torsional rigidity of the fold and on changes in the axial orientation of the flipon relative to other DNA segments [64–66,85,102].

## 6. The computational framework and a practical guide

By setting the energy required for a flipon to change conformation, the system is capable of learning from the output generated. As a result, a lower level of energy (i.e. less $\Delta E=w{\left(\Delta L\right)}^{2}$) is required to switch flipon conformations, while the probability of other alternative flipon structures is greatly diminished. Figure 3A gives one example of an assembly containing three fully connected flipons that initially fluctuate between the B- and Z-DNA conformations, with system noise affecting the overall probability of a flip. The noise is useful as it allows exploration of different combinations of flipon states, and the escape from suboptimal, local energy minima. Over time, the system is tuned to either minimize or maximize a particular output in response to intra- and extra-cellular inputs (setting flipons at each time point to either an idled ‘off’ (*p_s_ = 0*) or an active ‘on’ state (*p_s_ = 1*) (figure 3B). Conceptually, the tuning is a Bayesian process, with the current probability distribution of flipon states updated as new information becomes available. Overall, learning minimizes the sum of these transitions as represented by the min–max function of figure 3A. The learning rate is then given by δ(Δ*E*_s_)/δ*t* for each flipon in an ensemble. The adjustments improve future performance by resetting the threshold for assembling the appropriate cellular machinery at the locus where it is required. The tuning can involve only local flipons or incorporate others recruited from nearby or distant loci and can be summarized by how strongly the flipons states are coupled with each other. An increased number of active flipons in an ensemble allows both the response and the complexity of condensates to scale. The underlying phase transitions can then be set to operate close to criticality where a change in state propagates over a cellular domain [103].

Practically, the question arises of how to model such a computational framework? The implementation would take existing genomic maps of nucleosome-free regions defined by DNase I hypersensitivity sites (DHS). Greater than 94.5% of TF map to DHS [77], which are also enriched for experimentally validated G-, T- and Z-flipons [16,30,79]. The placement of the +1 nucleosome at transcription start sites and at the 3′ end of the DHS is strictly defined by the H3K4me3 (histone H3, trimethylated lysine 4) epigenetic mark [77]. Even a limited analysis based on the DHS maps reveals the power of this approach. Using just self-organizing maps, patterns spanning across multiple chromosomes and shared by different cell types and cell states were identifiable [77]. The inclusion of other measures will help better map nucleosome phasing and identify the open TF binding sites and those flipons free to cycle conformation. The available maps include those for protein-free (i.e. direct) RNA interactions with DNA and the different measures of chromatin contacts made with nucleosome resolution. The maps are based on techniques such as RADICL-seq to identify the small RNAs and long non-coding RNAs that potentially set flipon state [104], and Micro-C to capture changes in chromatin conformation and composition as cells switch state [105,106].

Perturbation data to train models is available from resources such as DepMap. The measures include those for gene, drug and microRNA disruptions [107–109]. Available validation sets include the single cell and tissue atlases of RNA expression, with measures for coding and non-coding transcripts and their modifications [110–112]. Models with a focus on how changes in flipon conformation alter RNA outputs are a good place to start. These models would take into account the interactions of flipons with RNAs, their impact on promoter usage, RNA expression and the epigenetic modifications that alter the subsequent processing of transcripts. Here the learning algorithm will optimize flipon settings that predict the active chromatin states involved. These studies could be initially restricted to a local topological domain where a perturbation creates a large effect (e.g. a heat shock response), or to contexts where cells transition to a new state (e.g. activation of a dormant cell, or induction of differentiation).

One interesting example to evaluate would be the sequential activation of genes during development within a *HOX* (homeobox protein) gene cluster [113,114]. Another equally interesting example is the initiation of the transition from maternal transcripts to zygote gene activation in 2-cell zygotes that depends on MTa (mouse transposon a) retroviral promoters. In this pathway, the MTa promoters iinitiate embryonic gene transcription, producing a chimeric transcript that drives the production of key cellular proteins. The transcript is made by splicing the retroviral and downstream RNAs together. Later, the MTa transcript undergoes cleavage rather than splicing, with the downstream gene promoter then driving cellular gene transcription [115]. Both the MTa retroelement splice and polyadenylation motifs are adjacent to each other, and both are emebedded within a cluster of potential triplex-forming flipons. The triplezes set by RNAs can position nucleosomes through their interactions with chromatin remodellers [116]. It is likely that the initial nucleosome placement favours splicing of the MTa transcript by ensuring that the splice site is in an open DNA region. Later, the nucleosomes are positioned to inhibit splicing and promote transcript cleavage at the polyadenylation site. In such an arrangement, inhibition of splicing and truncation of the retroviral transcript switches the transcription start site from the MTa promoter to the gene promoter. The transition is probably initiated by the spliced transcript made from the MTa promoter. This transcript folds back to form a triplex that changes the nucleosomal placement and the promoter used. Other examples of triplex-based promoter switches are known. For example, the SPHK1 downstream promoter produces an antisense KHPS1 RNA that initiates triplex formation at an upstream promoter, leading to enhanced expression of the sense transcript [117]. The human *β-globin* locus also uses a triplex switch to downregulate *ε-* and *γ-globin* upstream promoters and enhance expression of the downstream *β-globin* triplex-forming transcript [62]. These different use cases provide simple models for the evaluation of genomic learning models. In such examples, the probability that G- and Z-flipons in promoters adopt an alternative conformation is moderated by triplexes formed with long transcripts. Small RNAs targeting flipons and transposons, such as miRNA and piRNAs, or those induced by perturbation or sequestered at other sites, collectively adjust the probability that a particular flipon is active.

## 7. Flipons and p-bit computing

This overall schema represents an implementation of p-bit computing, a recently described architecture that offers greatly improved performance in estimating probability distributions compared with strictly deterministic digital computers, incurring a significantly lower energy cost and an easier implementation than possible with quantum bit systems [118]. An example given by Camsari and colleagues describes factorization of the number ‘*N*’. The approach uses an adder circuit composed of inherently noisy logic gates that randomly switch between ‘on’ and ‘off’ states. The adder is run in reverse to calculate the factors of ‘*N*’. The ‘off/on’ probability distribution of the adder gates undergoes tuning to minimize the difference between the current adder output and the target value. The reiteration terminates with the discovery of adder settings that generate *N*. The factors of *N* are then read from the adder registers [118]. The Camsari approach requires very fast clock times to ensure rapid convergence on the most likely solution. Further, these gates must be updated asynchronously to prevent resonance effects where gates cycle between a small number states without converging on a meaningful answer. Neither fast times nor precise wiring are features of biological systems.

The genomic implementations of p-bit computation also differ in a number of other ways from the Camsari implementation. There is a difference in scale, as genomes can exchange information over large distances. They communicate by directly altering the topology of chromosomal domains [119,120], or by local changes to DNA twist and writhe [121]. The outcomes may be less direct and mediated by the structure-specific docking of RNAs or involve architectural proteins that align distant DNA segments [122]. There are also limits to how fast flipons can switch states. The rate for Z-flipons is of the order of 100 ms [123], while G-flipons often represent energy minima that potentially trap the GQ conformation, with helicases and energy expenditure required to reform B-DNA [44,124]. The different kinetics are reflected in flipon biology; Z-flipons act as actuators, while G-flipons can serve as memory elements to preserve cell state during differentiation and cell division [53]. Flipons capable of adopting different alternative conformations increase the range of outcomes. The learning rate δ(Δ*E*_s_)/δ*t* then varies with the flipon involved.

## 8. Error correction by genomic systems

Another notable contrast between flipons, p-bit computers and Turing machines [125] is that halting by a biological system usually signifies failure, not success. To ensure continued operation, genomes implement robust error correction protocols (ECP) to triage and reduce the complexity of the output. Such ECP are necessary as circuits like those in figure 2C–F can produce a multitude of defective transcripts. Eliminating low-quality RNA transcripts that have splicing errors or premature stop codons by nonsense-mediated decay pathways improves system performance [126–128]. Other pathways make base modifications to nascent transcripts that direct the destruction of the RNA if they are not subsequently removed from the transcript. These marks, like methy-6-adenosine, are frqeuntly found in introns. A different set of ECPs use sequence-specific RNAs to guide the destruction of endogenous retroelements (ERE) and pathogen transcripts by piwi and argonaute proteins [67,129,130]. Other ECPs prevent flipons from permanently freezing in one state or another [131,132]. These pathways resolve persistent R-loops, facilitating the resolution of GQ by rehybridization of the G- and C-rich strands, or repair flipons when they break [84,133]. All these ECPs come at a metabolic cost as many of the RNA produced are never used. Instead, they are junked.

With the exception of *Monocercomonoides* species, eukaryotes depend upon mitochondria to power the setting and resetting of flipons that enable genomes to learn [134]. The strategy enables the rapid rewiring of genetic programmes without any change to the underlying genomic sequence while allowing genomes to learn those settings that are optimal for a particular set of conditions. The design provides a large canvas for evolutionary innovations that goes beyond the simple canalization of phenotypes [135]. Indeed, the evidence now suggests that canalization is easily reversed to regenerate stem cells [93]. The repetitive genome has a role to play in such processes: as the number of flipons increases, it becomes less likely that any two cells share the same exact flipon settings or have the same identical phenotype. In such cases, some cells are better adapted to the current context than others. The most attuned cells then are more likely to undergo selection and clonally populate tissues [136]. The outcomes may not represent the best of all possible solutions, but they allow individuals to survive the moment in ways that the selfish gene described by Dawkins does not [137].

## 9. Future

As with other disciplines, biology is moving to a more dynamic conceptualization of the processes occurring within the cell. As regulatory processes evolve, new adaptations are layered on top of pre-existing ones, with each subsequent innovation dependent on those that came before. Flipons probably represent an early form of genetic encoding that is still with us today and now provides a foundation for the more elaborate codon-based specification of the modern-day protein machinery (figure 1). Likewise, our understanding of such events is under constant revision. The changes are reflected in the way we currently conceptualize information flow within a cell. At the dawn of the electronic computer age, we looked for wiring diagrams that enabled the biology. Now the focus is on macromolecular condensates that coordinate critical cellular chemistries. The switches involved are the phase transitions that initiate condensate formation. The critical point reflects when and how the condensate formation is triggered. The current understanding is that the cell sets switches close to criticality. Similarly, the role of non-genetically encoded phase transitions in setting chromatin organization is more extensive than expected from bacterial genetic studies. The simpler genetic systems explored in the past were based on organisms with repetitive elements stripped from their genomes [138]. The use of host-based, rather than pathogen-targeted innate immunity is but one example we have learned of recently. In a subset of phyla, the rather haphazard retention of EREs evolved a highly programmable genome fashioned with flipons.

The Bayesian optimization of genomes is indeed empowered by the probabilistic nature of flipons. Despite their dependence on repetitive sequences, flipons increase the algorithmic complexity of genomes, all by tinkering with how the codon ciphers are parsed into programmes. Many of the concepts developed to further artificial intelligence by deep learning approaches, also have application to training genomes. This new knowledge will certainly improve our ability to engineer flipons and to reprogramme cells [139]. The models will empower us to design small molecules and RNA therapeutics that reset flipon conformations to prevent disease initiation and progression. The encoding of genetic information by flipons, while unexpected, is another example of how Nature exceeds our expectations.

## Data Availability

All data and methods are available or described in the main text or the references.
